# A pilot study on COVID-19 vaccine hesitancy among healthcare workers in the US

**DOI:** 10.1371/journal.pone.0269320

**Published:** 2022-06-15

**Authors:** Mofan Gu, Bruce Taylor, Harold A. Pollack, John A. Schneider, Nickolas Zaller

**Affiliations:** 1 Department of Health Behavior and Health Education, University of Arkansas for Medical Sciences Fay W. Boozman College of Public Health, Little Rock, Arkansas, United States of America; 2 National Opinion Research Center (NORC) at the University of Chicago, Chicago, Illinois, United States of America; 3 Crown Family School of Social Work, Policy, and Practice, The University of Chicago, Chicago, Illinois, United States of America; 4 Departments of Medicine and Public Health Sciences, The University of Chicago, Chicago, Illinois, United States of America; University of Bern, SWITZERLAND

## Abstract

To explore the attitude towards COVID-19 vaccination among healthcare workers in the US, we surveyed three groups of individuals (essential non-healthcare workers, general healthcare workers, and correctional healthcare workers). We found surprisingly high portions of healthcare workers with COVID-19 vaccine hesitancy/resistance, with 23% of correctional healthcare workers and 17% general healthcare workers (as compared to 12%) refusing to be vaccinated against COVID-19. Multivariate regression models suggest that current season flu vaccination (aOR = 3.34), relying on employer for COVID-19 information (aOR = 3.69), and living in the Midwest (aOR = 5.04) to be strongly associated with COVID-19 vaccine acceptance among essential workers and general healthcare workers. Current season flu vaccination (aOR = 7.52) is also strongly associated with COVID-19 vaccine acceptance among correctional healthcare workers. Potential mechanisms of vaccine hesitancy/resistance among healthcare workers involves low health literacy and employer mistrust. Our findings are highly relevant as we try to reach COVID-19 vaccination goals in the US.

## Introduction

Coronavirus disease (COVID-19) is an infectious disease caused by the SARS-CoV-2 virus [1]. On March 11, 2020, the World Health Organization (WHO) declared the COVID-19 outbreak a global pandemic [1]. Since the first confirmed COVID-19 case in the US in January 2020, the virus has led to 48.2 million confirmed cases and 776,536 deaths in the US [2]. The COVID-19 pandemic has disproportionally impacted marginal population groups including the elderly, people of color, low-income households, individuals with criminal justice involvement, and many others [3–5]. In particular, jails and prisons have been hit hard by the pandemic. Some 20% of all individuals incarcerated in state and federal prisons have tested positive for COVID-19, a rate more than four times as high as that in the general population [6]. Across the US, more than 440,000 prisoners have been infected with COVID-19, with over 2,600 deaths, with another close to 123,300 staff working in prisons testing positive for COVID-19 with 242 deaths of correctional personnel [7]. Additionally, people who are incarcerated face three times the risk of death from COVID-19 [8]. By August, 2020, 90 of the largest 100 cluster outbreaks in the US occurred in prisons and jails [7].

A growing body of literature shows that COVID-19 vaccines are safe and effective [9]. COVID-19 vaccines reduce likelihood of infection and severe complications [9]. The benefit of COVID-19 vaccines outweigh the risk of rare adverse events [9]. On December 11 and December 18, 2020, the Food and Drug Administration (FDA) issued emergency use authorization (EUA) for the Pfizer-BioNTech COVID-19 Vaccine and Moderna COVID-19 vaccine [1]. On February 27, 2021, the FDA issued EUA for the Johnson & Johnson single-dose COVID-19 Vaccine [1]. Various public health agencies, including the WHO and US Centers for Disease Control and Prevention (CDC), have recommended COVID-19 vaccines to all eligible individuals [9]. As of November 2021, 68.8% of the US population has received at least one dose of COVID-19 vaccine, and 59.1% are fully vaccinated [2].

Despite the clear and documented benefits of COVID-19 vaccines, there continues to be resistance to vaccination among many individuals in the US, including health care workers. Reasons for vaccine hesitancy include vaccine-specific concerns (safety and effectiveness), need for more evidence/information, antivaccine beliefs, and lack of institutional trust [5]. Several studies have assessed factors associated with COVID-19 vaccine hesitancy. Common factors include low educational attainment, ethnic differences, rurality, and resistance towards other vaccinations (e.g., influenza) [5, 10]. However, the evidence is still evolving with respect to the most significant predictors of being resistant to COVID-19 vaccination. While COVID-19 vaccination hesitancy rates in the general population have been explored comprehensively and are fairly well established, few studies have specifically explored COVID-19 vaccine hesitancy/reluctance among health care workers [10–12]. Some data suggests healthcare workers are also hesitant about receiving the COVID-19 vaccine [13–19]. The situation is especially concerning in correctional facilities. Despite the governments’ effort to prioritize correctional staff in vaccination schedules, the vaccination rate remained fairly low—as of April 2021, the median correctional staff vaccination rate across the US was 48% [20, 21].

Therefore, in this exploratory pilot study, we aimed to assess correctional health care workers,’ general healthcare workers’ and essential workers’ willingness to receive, refuse or express hesitancy towards the COVID-19 vaccine; reasons for getting or not getting the COVID-19 vaccine; and sources of COVID-19 information. In addition, we compare our results from the correctional health care staff to healthcare workers in non-jail environments and general essential workers over the COVID-19 pandemic (both drawn from a general population survey of US households). These results are designed to provide data for developing a larger study of a representative sample of correctional health care staff in multiple regions of the US.

## Methods

### Surveys

This exploratory study includes a cross-sectional random sample of participants drawn from two main sources. The first sample (n = 78) was a group of healthcare professional (mostly nurses) from 65 local jail facilities across eight states (OK, AR, TX, LA, KS, MO, NM, CO). All eligible jail healthcare professional staff (mostly nurses) were offered the survey and 80% completed the survey. The research team coordinated the distribution of the survey with the medical director of the health care staff. Potential participants, all adults, were emailed a link to the online survey by the medical director. The second data source was AmeriSpeak^®^, a probability-based ongoing panel of about 35,000 households designed to be representative of the US household population. Over the same general time frame (spring of 2021) as the jailed-based health care worker survey, we selected all general healthcare workers (n = 65) and a random sample of self-reported essential workers (n = 100) during the COVID-19 pandemic but outside of the health field from AmeriSpeak’s ongoing panel of surveys. All general healthcare workers had either “healthcare practitioner and technical occupations” or “healthcare support occupations,” and reported patient contact during work.

For the AmeriSpeak^®^ panel, a stratified random sample of U.S. households are selected and sampled using area probability and address-based sampling, with a known, nonzero probability of selection from the NORC at the University of Chicago (NORC) National Sample Frame. These sampled households are then contacted by U.S. mail, telephone, and field interviewers (face-to-face) to capture harder to reach cases. The panel provides sample coverage of approximately 97% of the U.S. household population [22] and compares favorably to the US Census American Community Survey [23, 24]. The annual panel retention rate is about 85% [22]. AmeriSpeak panel’s weighted household recruitment rate is 37%, one of the highest for comparable national probability-based household panels [25]. The AmeriSpeak panel implements monthly Omnibus surveys using a probability sample of adults.

For this study, our team implemented our survey under AmeriSpeak’s monthly Omnibus survey program. AmeriSpeak staff sent an email to a randomly-selected group of panel members describing the study, covering informed consent and inviting them to participate in the survey. One-quarter of the contacted participants from the AmeriSpeak panel of 3,900 invited adult panelists completed this project’s survey (n = 1,161). For this study, we selected all general healthcare workers (n = 65) and a random sample of essential workers (n = 100) during the COVID-19 pandemic but outside of the health field from AmeriSpeak to participate in this study.

Participation in the survey was strictly voluntary and informed consent was required before a participant was allowed to work on a survey. The study was approved by one of the author’s organization’s Institutional Review Board (with a multiple project assurance with the U.S. Department of Health and Human Services) for the conducting of human subject’s research. All survey participants completed the survey in English. Sample participants who did not respond to the initial invitation were contacted about three to four times by email to complete the survey.

Medical staff responsible for providing healthcare in a jail environment across eight states were invited to complete an 85-item survey on perceptions of COVID-19 related risk, experiences with COVID-19 risk mitigation strategies, and willingness to participate in COVID-19 vaccination campaigns from the end of March 2021 to May 31, 2021. Next, a cross-sectional sample of AmeriSpeak participants completed surveys during a data collection period from February 10, 2021 to March 22, 2021 (n = 1,161 participants). Participants were given a subset of 40 of the same 85-item survey items covering COVID-19 protective behavior including vaccinations and demographic/background questions. The survey items in common for the two samples were used in this paper (Table 1).

**Table 1:** Sample characteristics and vaccination status.

Survey	AmeriSpeak Essential Workers	AmeriSpeak Healthcare Workers	Jail-based Healthcare Workers
**N**	100	65	78
**Age** *			
• 18–29	22 (22%)	11(17%)	5 (8%)
• 30–44	40 (40%)	23 (35%)	29 (46%)
• 45–59	29 (29%)	25 (38%)	25 (40%)
• 60+	9 (9)	6 (9%)	4 (6%)
**Sex**			
• Male	52 (52%)	21 (32%)	12 (15%)
• Female	48 (48%)	44 (68%)	66 (85%)
**Race**			
White	55 (55%)	42 (65%)	63 (81%)
Black	15 (15%)	8 (12%)	4 (5%)
Other	30 (30%)	15 (23%)	11 (14%)
**Region**			
• Northeast	9 (9%)	9 (14%)	0
• Midwest	32 (32%)	24 (37%)	2 (3%)
• South	27 (27%)	12 (18%)	61 (78%)
• West	32 (32%)	20 (31%)	5 (6%)
• Multiple	0	0	10 (13%)
**Household income**			
• 50,000 or less	28 (29%)	23 (35%)	15 (19%)
• 50,001–100,000	42 (43%)	23 (35%)	29 (37%)
• Over 100,000	27 (28%)	19 (29%)	25 (32%)
**Education**			
• High School or less	6 (6%)	2 (3%)	0
• Some college / College graduate	81 (81%)	49 (75%)	52 (67%)
• More than college	13 (13%)	14 (22%)	26 (33%)
**Flu Vaccination**			
• Ever vaccinated	80 (80%)	51 (78%)	78 (100%)
• Vaccinated this season	62 (62%)	44 (68%)	45 (58%)
**COVID-19 Vaccination**			
• Already vaccinated	15 (15%)	35 (54%)	51 (65%)
• Not vaccinated but will ASAP	37 (37%)	3 (5%)	1 (1%)
• Will wait	22 (22%)	11 (17%)	0
• Will not be vaccinated	12 (12%)	11 (17%)	18 (23%)
• Not sure / Refused to answer	14 (14%)	5 (8%)	8 (11%)

*missing = 15 (all from jail-based healthcare worker survey)

### Measures

Survey items covered the nature of the participants’ work, work environment, COVID-19/flu vaccination history and vaccination hesitancy, reasons for getting or not getting the COVID-19 vaccine, and sources of COVID-19 information. We also included a series of demographic and background questions such as age, biological sex at birth (male or female), region of US the person works within (Northeast, Midwest, South, West or multiple regions), and education (high school or less, some college, college graduate and more than college).

#### Vaccine willingness/acceptance

Our main outcome of interest was willingness to be vaccinated against COVID-19. In the surveys, participants reported their COVID-19 vaccine status. To be considered “willing”, a participant had to be either already vaccinated (one dose or more), or willing to be vaccinated as soon as possible. If a participant expressed disapproval or reluctance of receiving the vaccine, they were considered “unwilling”.

#### Reasoning for vaccine hesitancy/resistance

In addition to vaccine willingness, we also asked for reasons why individuals chose to be vaccinated or not. For those unvaccinated participants, we also asked participants about factors that would make them reconsider being vaccinated. Participants could report more than one reason/factor.

#### Flu vaccination

The first covariate was flu vaccination status. Participants were asked whether they were ever vaccinated against seasonal flu, and whether they had been vaccinated for the current flu season.

#### Source of information

Each participant was asked where they obtained most of their information on COVID-19 within the past month. Sources of information included newspaper (local and national print), television (broadcast and cable), online news, social media, friends and family, employer, government (federal and state), scientific journals, and other sources (e.g., radio, church, and community-based organizations). Participants could report more than one source of information.

#### COVID-related deaths

We also asked participants whether or not anyone they knew had died from COVID-19, including people within the participants’ household, friends, family members, acquaintances, and coworkers.

#### Demographics

Lastly, we collected the participants’ demographic information, including current age, sex, state of residence, and education level.

### Statistical analysis

#### Descriptive analysis

We first assessed the distribution of COVID-19 vaccine status, and associated reasons for vaccine willingness or refusal. We compared the distribution across all three groups, as well as within the two AmeriSpeak groups, using chi-square tests. We repeated the analysis with sources of COVID-19 information.

#### Logistic regression

To assess factors contributing to vaccine willingness, we constructed multivariable logistic regression models. The outcome was the dichotomous variable of vaccine willingness (yes/no), and we included several covariates which potentially influence COVID-19 vaccination acceptance in the model, including flu vaccination status, source of COVID-19 information, and demographic variables (current age, biological sex, region of residence, educational attainment). We first tested the model within the correctional healthcare group. Then we tested the model within the two AmeriSpeak groups (essential non-healthcare workers, and general healthcare workers), with employment type (group assignment) as an additional covariate. Coefficients were exponentiated to obtain adjusted odds ratios (aOR).

## Results

### Sample characteristics

Participant demographics are shown in Table 1. Among participants in all three samples, the age range was from 18 to about 60 years old. The essential worker and jail-based healthcare worker samples had a large proportion of female respondents relative to the general health care workers (68% and 85%, respectively, versus 48%). Most participants in all three samples self-identified as White, non-Hispanic, and most participants had at least some college or more education. Income was similar across all three samples with the jail-based healthcare workers having a slightly higher average income (Table 1).

Overall, 15% of essential non-healthcare workers, 54% of general healthcare workers, and 65% of correctional healthcare workers reported receiving their COVID-19 vaccination (Table 1). Among the total sample (all three groups), 12% of essential non-healthcare workers, 17% of general healthcare workers, and 23% of correctional healthcare workers reported that they will not consider COVID-19 vaccination (Table 1). A large portion (37%) of essential non-healthcare workers expressed willingness to be vaccinated against COVID-19 as soon as possible even though they had not yet been vaccinated.

### Descriptive analysis findings

#### Reasoning behind COVID-19 vaccine hesitancy/resistance

Among participants in all three samples who reported not willing to receive a COVID-19 vaccine, common reasons included potential side effects, not being concerned about becoming seriously ill from COVID and COVID-19 vaccines being promoted by politicians to win votes without sufficient testing. Among general healthcare workers, another common reason participants endorsed for not being vaccinated against COVID-19 was the belief that the COVID-19 pandemic is not as severe as many people think. A greater proportion of participants in the general healthcare worker group and in the jail-based healthcare worker group indicated that nothing would make them reconsider being vaccinated against COVID-19 (63% and 67%, respectively). In contrast, only 19% of the essential non-healthcare workers expressed a similar unwillingness to reconsider their vaccination status.

#### Source of COVID-19 information

The main sources of COVID-19 information, stratified by COVID-19 vaccination status and sample group, are shown in Table 2.

**Table 2:** Sources of COVID-19 information.

	Already vaccinated	Will ASAP	Will wait	No	Not sure / refused
**AmeriSpeak Essential Workers**					
• Government	73%	59%	45%	25%	43%
• TV	47%	70%	73%	50%	29%
• Online news	40%	32%	23%	42%	36%
• Personal network	20%	38%	27%	33%	50%
• Social media	33%	35%	27%	50%	29%
**AmeriSpeak Healthcare Workers**					
• Government	60%	33%	18%	36%	60%
• Employer	60%	33%	9%	36%	20%
• TV	49%	33%	45%	36%	40%
• Online news	43%	33%	27%	18%	20%
• Personal network	31%	67%	45%	18%	20%
• Social media	31%	33%	27%	9%	20%
**Jail-based Healthcare Workers**					
• Government	55%	0%	NA	39%	50%
• Employer	55%	0%	NA	72%	50%
• Scientific journal	37%	100%	NA	44%	0%
• TV	35%	0%	NA	50%	38%
• Social media	12%	0%	NA	39%	38%

*Participants may report more than one sources.

** Only the top 3 sources were included in the table.

### Factors associated with COVID-19 vaccine willingness/acceptance

Table 3 provides multivariable logistic regression results (with an alpha cut-off level of 0.05 for statistical significance) for key factors associated with COVID-19 vaccine willingness.

**Table 3:** Factors associated with COVID-19 vaccine willingness, based on logistic regression (full models and sensitivity analyses).

	*Full Analyses*			*Sensitivity analyses* *		
	Adjusted Odds Ratio**	95% Confidence Interval	p	Adjusted Odds Ratio**	95% Confidence Interval	p
***AmeriSpeak Sample***						
• Influenza vaccination						
○ Current^§^	**3.34**	**(0.98, 11.49)**	**0.05**			
○ Ever	4.31	(0.93, 20.0)	0.06			
• COVID-19 information source						
○ Employer^§^^†^	**3.69**	**(1.21, 11.24)**	**0.02**	**3.27**	**(1.22, 8.72)**	**0.02**
○ Newspaper	1.70	(0.49, 5.88)	0.40	1.77	(0.59, 5.34)	0.31
○ TV	1.16	(0.46, 2.94)	0.76	1.02	(0.44, 2.36)	0.96
○ Government	1.48	(0.61, 3.58)	0.39	1.73	(0.77, 3.88)	0.19
○ Online news	0.77	(0.28, 2.07)	0.60	0.77	(0.31, 1.88)	0.56
○ Social media	1.59	(0.59, 4.31)	0.36	1.55	(0.62, 3.86)	0.35
○ Personal network	0.81	(0.28, 2.36)	0.71	0.73	(0.29, 1.88)	0.52
○ Scientific journal	3.45	(0.59, 20.41)	0.17	3.35	(0.66, 17.07)	0.15
○ Other	1.91	(0.58, 6.33)	0.29	1.81	(0.60, 5.41)	0.29
• Knowing someone who died from COVID-19^†^	1.82	(0.74, 4.48)	0.19	**2.57**	**(1.14, 5.80)**	**0.02**
• Gender (male vs. female)	1.07	(0.46, 2.53)	0.87	1.01	(0.47, 2.18)	0.98
• Age						
○ 18–29	reference			reference		
○ 30–44	1.58	(0.47, 5.39	0.51	1.90	(0.62, 5.77)	0.57
○ 45–59	1.34	(0.34, 5.24)	0.34	1.74	(0.51, 5.90)	0.46
○ 60 and above^†^	8.02	(0.99, 64.93)	0.06	**8.69**	**(1.52, 49.53)**	**0.03**
• Race						
○ Black	reference			reference		
○ White	3.12	(0.85, 11.46)	0.17	2.75	(0.82, 9.18)	0.17
○ Other	2.40	(0.58, 10.01)	0.59	2.09	(0.56, 7.87)	0.66
• Education						
○ Less than high school	reference			reference		
○ Some/full college	2.93	(0.29, 30.05)	0.74	3.80	(0.51, 28.21)	0.30
○ More than college	5.38	(0.41, 70.89)	0.19	4.05	(0.44, 37.49)	0.33
• Region of residence						
○ South	reference			reference		
○ Northeast	0.66	(0.15, 2.85)	0.09	0.80	(0.20, 3.20)	0.25
○ Midwest^§^^†^	**5.04**	**(1.55, 16.41)**	**0.002**	**3.19**	**(1.15, 8.89)**	**0.01**
○ West	1.67	(0.50, 5.60)	0.84	1.48	(0.51, 4.33)	0.87
• Occupation (health worker vs. essential)	1.07	(0.42, 2.76)	0.88	1.05	(0.45, 2.43)	0.92
***Jail-based Healthcare Worker Sample***						
• Influenza vaccination						
○ Current^§^	**7.52**	**(1.13, 50.0)**	**0.04**			
○ Ever						
• COVID-19 information source						
○ Employer	2.37	(0.25, 22.73)	0.45	1.64	(0.24, 11.23)	0.62
○ Newspaper	0.16	(0.01, 2.75)	0.21	0.16	(0.01, 2.22)	0.17
○ TV	0.46	(0.05, 4.44)	0.50	0.30	(0.04, 2.42)	0.26
○ Government	3.52	(0.34, 35.71)	0.29	3.44	(0.45, 26.31)	0.23
○ Online news	0.50	(0.04, 7.04)	0.61	0.66	(0.07, 6.68)	0.72
○ Social media	0.56	(0.02, 13.70)	0.72	0.36	(0.03, 5.13)	0.45
○ Personal network	1.64	(0.06, 43.48)	0.77	3.04	(0.15, 61.60)	0.47
○ Scientific journal	0.23	(0.02, 2.54)	0.23	0.38	(0.05, 2.86)	0.35
○ Other	0.49	(0.02, 9.71)	0.64	0.65	(0.03, 14.29)	0.78
• Knowing someone who died from COVID-19	2.25	(0.33, 15.38)	0.41	1.53	(0.27, 8.77)	0.64
• Gender (male vs. female)	2.01	(0.06, 71.35)	0.70	1.31	(0.06, 27.56)	0.86
• Age						
○ 18–29	reference			reference		
○ 30–44	1.16	(0.10, 13.37)	0.95	1.15	(0.09, 14.49)	0.94
○ 45–59	0.63	(0.04, 10.02)	0.94	1.25	(0.09, 18.00)	0.95
• Education						
○ Some/full college	reference			reference		
○ More than college^†^	7.58	(0.76, 76.92)	0.08	**9.97**	**(1.18, 84.25)**	**0.03**

* Sensitivity analyses included all variables in the full models except for influenza vaccination.

** Outcome is willingness for COVID-19 vaccination (willing compared to not willing)

^§^ Statistically significant (based on an alpha = 0.05 cut-off) in the full models

^†^ Statistically significant (based on an alpha = 0.05 cut-off) in the sensitivity analyses

In the AmeriSpeak sample (essential non-healthcare workers and general healthcare workers), flu vaccination in the current season was associated with willingness to receive COVID-19 vaccinations. Those vaccinated for flu were three times as likely to be willing to receive COVID-19 vaccines, as compared to those not vaccinated for flu in the current season (aOR, 3.34; 95% CI, 0.98, 11.49). Participants who obtained most of their COVID-19 information from their employers in the past month were almost 4 times as likely to be willing to receive COVID-19 vaccination, as compared to those who didn’t obtain COVID-19 information from their employer (aOR, 3.69; 95% CI, 1.21, 11.24). In addition, participants from the Midwest were 5 times as likely to be willing to receive COVID-19 vaccination, as compared to participants in the South (aOR, 5.04; 95% CI, 1.55, 16.41).

In the jail-based healthcare worker sample, flu vaccination in the current season was the only factor significantly associated with willingness to receive COVID-19 vaccinations. Those vaccinated for flu were over seven times as likely to be willing to receive COVID-19 vaccines, as compared to those not vaccinated for flu in the current season (aOR, 7.52; 95% CI, 1.13, 50.0).

### Sensitivity analyses

Considering how strong influenza vaccination is associated with COVID-19 vaccine willingness, we conducted sensitivity analyses where we ran the regression models without influenza vaccination variables. After omitting influenza vaccination, several other factors become statistically significant (Table 3). Among essential non-healthcare workers and general healthcare workers, knowing someone who passed from COVID-19, and being 60 and older are significantly associated with being willing to receive COVID-19 vaccination (aORs 2.58 and 8.69 respectively), in addition to relying on employer on COVID-19 information and living in the Midwest which we observed in the original model. Among jail-based healthcare workers, having educational attainment higher than college is significantly associated with COVID-19 vaccine willingness (aOR 9.97).

## Discussion

Healthcare workers are at higher risk of COVID-19 infection because of continuous exposure to patients. It is crucial for healthcare workers to achieve high COVID-19 vaccination rates for the protection of themselves and for people, especially patients, around them. Healthcare workers in correctional settings are an especially important population to be vaccinated, because they work in closed environments filled with extremely vulnerable populations (e.g., individuals with high prevalence of chronic diseases). The risk is further exacerbated by poor conditions within correctional facilities (overcrowding, poor ventilation, etc.) and high turnover rates of people who are incarcerated in jail settings [26]. COVID-19 case rates usually grow exponentially once the virus is introduced to a correctional facility, impacting both individuals who are incarcerated and correctional healthcare workers. Correctional facilities are a key source for COVID-19 cases in the US. According to public data, COVID-19 case rates among inmates and correctional staff have grown consistently higher than those in the general population since March 2020 [26–28]. By the end of November 2021, prisons in the US publicly reported 123,294 staff members that tested positive for COVID-19, and 242 related staff deaths [7]. Due to lack of systematic testing, correctional healthcare workers may bring the virus into their workplace and back out to their homes in the community, further fueling community transmission [28]. In addition, healthcare workers could play a significant role in influencing vaccine readiness in the general public. Healthcare workers are frequently looked to as important sources of reliable information—high vaccination rate among healthcare workers could potentially increase vaccination rates in the general public [29]. Therefore, vaccination of healthcare workers is potentially a key in COVID-19 infection prevention and control [30].

There were significantly more healthcare workers in our sample, as compared to essential non-healthcare workers, who have already received vaccines for COVID-19. However, a larger portion of healthcare workers (23% for correctional healthcare workers and 17% for general healthcare workers) reported that they would not consider getting the COVID vaccine, as compared to essential workers (12%); surprisingly, the majority of healthcare workers who refused to be vaccinated stated that nothing would make them reconsider COVID-19 vaccines (67% among correctional healthcare workers and 63% among general healthcare workers, as compared to 19% among essential workers).

The main reason for not vaccinating, across all three groups, were potential side effects (73%-94%). All three COVID-19 vaccines in the US, approved by the Food and Drug Administration, have been proven safe and effective against severe COVID-19 infections [31]. Millions of Americans have been safely vaccinated, and only rare cases of severe side effects have been reported [31]. Most often, side effects include mild symptoms like pain, swelling, muscle ache and fatigue [31]. It is important to provide proper context for fear of potential adverse side effects from vaccinations and to educate everyone, especially healthcare workers, that from a public health perspective and an individual health perspective, the benefits of vaccines outweigh the risk of rare/mild side effects. By choosing not to vaccinate, people are susceptible to severe COVID-19 illness and could potentially lose their lives or become severely ill and have to be out of work for a significant period of time. Furthermore, unvaccinated individuals can potentially transmit COVID-19 to more vulnerable patient populations in healthcare and/or correctional settings.

In addition to potential side effects, many unvaccinated healthcare workers were not concerned about getting severely ill from COVID-19 (25% in general healthcare workers, and 43% for correctional healthcare workers); and approximately one-third of general healthcare workers and essential workers believed that COVID-19 vaccines were being promoted by politicians only to win election votes even when these vaccines were not sufficiently tested for effectiveness or safety. These findings go hand-in-hand with skepticism about COVID-19 vaccine safety/effectiveness. Some healthcare workers in our sample believe that vaccines are more harmful than COVID-19, despite witnessing all the illness and deaths at work from COVID-19. These concerns need to also be incorporated into education and messaging about COVID-19 vaccines in order to dispel misinformation. In addition, there are two potential factors that may be highly influential: health literacy and employer trust.

Health literacy is defined as “the degree to which individuals and groups can obtain process, understand, evaluate, and act upon information needed to make public health decisions that benefit the community” [32]. When a person lacks health literacy, they may be more susceptible to misinformation and mistrust, which are known to contribute to vaccine hesitancy [33–35]. Despite common perceptions, health literacy among healthcare workers could vary significantly, considering that “healthcare workers” include a wide range of positions from physicians to caretakers. It is crucial to improve health literacy in all healthcare workers within the context of the COVID-19 pandemic, so they can better understand, process, and critique the vast amount of urgent, complex and sometimes conflicting information from various sources [36]. One important way to boost health literacy would be to identify/use reliable sources for COVID-19 information, such as resources from the CDC. Based on our study, those who reported being willing to be vaccinated were more likely to rely on trustworthy sources like the government and employers, rather than sources like social media, online news, and personal networks (see Table 2). Social media and online news are infamous for spreading of vaccine information due to the lack of editorial curation and scientific vetting [37]. Anyone with access to the internet could post personal experiences/opinions on social media. These posts, frequently employ vivid narratives and powerful imagery, and can reach large audiences rapidly [37]. In addition, education level was significantly associated with COVID-19 vaccine willingness among jail-based healthcare workers in our sample (Table 3). Healthcare workers should be provided with additional trainings to use more validated and verified sources and to evaluate critically health information before making public health decisions. Moreover, our findings underscore the importance of accurate Covid-19-related information, especially on social media platforms and online news sites. It is crucial to ensure transparent reporting of vaccine information in a manner that people of all educational levels can understand, and engage more target audiences with narratives/stories (e.g., personal experiences in the ER/ICU, stories of family members who passed away from COVID-19) [33]. This is supported by our regression model, where COVID-19 vaccine willingness increased when the individual knows someone who passed from COVID-19 (Table 3).

In our study, the majority of correctional healthcare workers relied on their employer for COVID-19 information, including those refusing to be vaccinated. We suspect some level of employer mistrust leads to vaccine hesitancy among correctional healthcare workers. Employer mistrust has long existed in correctional settings due to understaffing, low pay, high workload, and many other factors [38]. Such mistrust has only worsened during the COVID-19 pandemic, when correctional facilities have often failed to establish adequate protocols to protect healthcare workers and other correction staff from COVID-19. In many states, correctional facilities do not systematically screen employees for COVID-19 infections, and employees are not universally guaranteed medical benefits or paid sick leave [27, 39]. Employees may be reluctant to be vaccinated because they cannot take any time off work if they experience any side effects. These poor occupational health standards potentially fuel employer mistrust in correctional facilities, and may contribute to vaccine hesitancy among correctional healthcare workers. This is further supported by our regression analyses, where relying on an employer for COVID-19 information is a significant predictor of COVID-19 vaccine willingness. Employers are essential in delivering vaccine messages, which could be optimized when there is trusting working environment that safeguards employers against COVID-19.

Based on our regression analyses, current influenza vaccination is also a key predictor of COVID-19 vaccination willingness—those who were currently vaccinated against influenza were 3 to 7 times more likely to be willing to receive COVID-19 vaccines, as compared to those who were not currently vaccinated against influenza. Up-to-date influenza vaccination may be an indicator of higher health literacy [40]. In future studies, influenza vaccine acceptance could serve as a proxy for COVID-19 vaccine acceptance and help to make predictions about willingness to receive a COVID-19 vaccine. Better understanding factors related to influenza vaccine acceptance could help to create tailored and targeted messages to reach high risk populations that are not yet vaccinated against COVID-19 [33]. Influenza vaccination strategies could also be used as case studies to help close disparities in vaccine uptake and to further explore reasoning behind vaccine acceptability and/or hesitancy.

### Limitations

The study has several limitations. First, the sample size is relatively small (especially the healthcare worker samples—65 general and 78 correctional). The sample is also fairly homogeneous (e.g., the correctional healthcare workers were 80% White) which limits our ability to examine effectively demographic factors as predictors for vaccine willingness. Second, the study uses two existing cross-sectional surveys, and we were limited to data collected by the survey. There are potentially unadjusted confounders in our regression models. Third, the study faces the usual limitations of relying on self-reports from surveys (e.g., respondents not accurately recalling their behavior or the basis for their beliefs) and future research should consider integrating medical records on actual vaccine acceptance to the research.

## Implications and conclusion

This study has several policy implications. First, our data indicate the significance of accurate and transparent COVID-19 information, especially on social media platforms and online news sites. This aligns with the CDC’s emphasis on finding credible vaccine information—it is crucial for the general public, as well as health care workers, to be exposed to credible and accurate COVID-19 information, in order to make informed healthcare decisions [41]. Second, our results may help to inform revised policies within correctional facilities. Improving working conditions, implementing adequate COVID-19 strategies, and strengthen employer-employee communications could potentially benefit vaccine uptake in the long run.

In this exploratory study, we surveyed three groups of individuals (essential non-healthcare workers, general healthcare workers, and correctional healthcare workers) on their attitude towards COVID-19 vaccination. We found surprisingly high portions of healthcare workers with vaccine hesitancy/resistance. We also found that flu vaccination, source of COVID-19 information, and region of residence to be strongly associated with COVID-19 vaccine acceptance. Potential mechanisms of vaccine hesitancy/resistance among healthcare workers may involve low health literacy and employer mistrust. Findings of this study inform a follow-up larger study to explore the merits of recommending policy changes including better COVID-19 information dissemination and improvement of working conditions at correctional facilities.

## Supporting information

S1 File Survey deidentified: De-identified survey dataset and codebook. (XLSX)
